# The Bioconductor channel in *F1000Research*

**DOI:** 10.12688/f1000research.6758.2

**Published:** 2016-04-20

**Authors:** Wolfgang Huber, Vincent Carey, Sean Davis, Kasper Daniel Hansen, Martin Morgan

**Affiliations:** 1 EMBL, 69117 Heidelberg, Germany; 2 Channing Division of Network Medicine, Brigham and Women's Hospital and Harvard Medical School, Boston, MA, 02115, USA; 3 Center for Cancer Research, National Cancer Institute, National Institutes of Health, Bethesda, MD, 20892, USA; 4 McKusick-Nathans Institute of Genetic Medicine, Johns Hopkins University, Baltimore, MD, 21205, USA; 5 Computational Biology Program, Fred Hutchinson Cancer Research Center, Seattle, Washington, USA

**Keywords:** Bioconductor, bioinformatics, genomics, systems biology, R, statistical computing

## Abstract

Bioconductor ( bioconductor.org) is a rich source of software and know-how for the integrative analysis of genomic data. The Bioconductor channel in F1000Research provides a forum for task-oriented workflows that cover a solution to a current, important problem in genome-scale data analysis. It also hosts manuscripts describing software packages, package-based vignettes, teaching labs, benchmark studies, methodological reviews and bioinformatics software oriented perspective papers.

## Editorial

Bioinformatics is a wonderful field, since so many of the researchers who develop new concepts, methods and tools have embraced the sharing of their research outputs as free, open-source software. This openness has, no doubt, accelerated the breathtaking progress in the field of genomics (and related `omic technologies). Bioconductor ( bioconductor.org) has become a place that many developers are choosing to host their software - it gives them a useful infrastructure for development and for providing effective user support ^1^. It is also a venue where many prospective users go to when they look for a quality solution in genome-scale data analysis, where they will find some of the most advanced tools.

This abundance of tools, however, can be difficult to navigate. Bioconductor comes in two parts: as a collection of individual packages, each developed independently by a research group somewhere on the globe, and often among the experts in their field; and as a layer of infrastructure, with common data structures and core library functionality to glue the packages together and provide, as we call it, ‘interoperability’.

The Bioconductor channel in F1000Research hosts **task-oriented workflows**. Each workflow covers a solution to a current, important problem in genome-scale data analysis from end to end. Often it will invoke resources from several packages; combine multiple data types and demonstrate integrative analysis and modelling techniques. We explicitly welcome workflows that are written by authors that not the developers of any of the used packages.

Of course we also welcome **package descriptions** and **package-based vignettes** by package authors that show how their package(s) can be employed to solve a scientific question, either by itself or in conjunction with other Bioconductor packages.

The channel also welcomes bioinformatics teaching labs, benchmark studies, methodological reviews and bioinformatics software oriented perspective papers.

We encourage the use of Rmarkdown and knitr (or similar facilities) for authoring papers in this channel. By integrating concrete software examples with textual motivation and explanation this lets the papers be alive.

We are excited about the rapid and transparent publication process of F1000Research and look forward to community contributions.
